# The Curious Case of Arenavirus Entry, and Its Inhibition

**DOI:** 10.3390/v4010083

**Published:** 2012-01-13

**Authors:** Jack H. Nunberg, Joanne York

**Affiliations:** Montana Biotechnology Center, The University of Montana, Missoula, MT 59812, USA; Email: joanne.york@umontana.edu

**Keywords:** arenavirus, envelope glycoprotein, fusion protein, fusion inhibitor, pH-dependent membrane fusion, endosome, antiviral, zinc binding, stable signal peptide, hemorrhagic fever

## Abstract

Arenaviruses comprise a diverse family of enveloped negative-strand RNA viruses that are endemic to specific rodent hosts worldwide. Several arenaviruses cause severe hemorrhagic fevers in humans, including Junín and Machupo viruses in South America and Lassa fever virus in western Africa. Arenavirus entry into the host cell is mediated by the envelope glycoprotein complex, GPC. The virion is endocytosed on binding to a cell-surface receptor, and membrane fusion is initiated in response to physiological acidification of the endosome. As with other class I virus fusion proteins, GPC-mediated membrane fusion is promoted through a regulated sequence of conformational changes leading to formation of the classical postfusion trimer-of-hairpins structure. GPC is, however, unique among the class I fusion proteins in that the mature complex retains a stable signal peptide (SSP) as a third subunit, in addition to the canonical receptor-binding and fusion proteins. We will review the curious properties of the tripartite GPC complex and describe evidence that SSP interacts with the fusion subunit to modulate pH-induced activation of membrane fusion. This unusual solution to maintaining the metastable prefusion state of GPC on the virion and activating the class I fusion cascade at acidic pH provides novel targets for antiviral intervention.

## 1. Introduction

**Figure 1:**
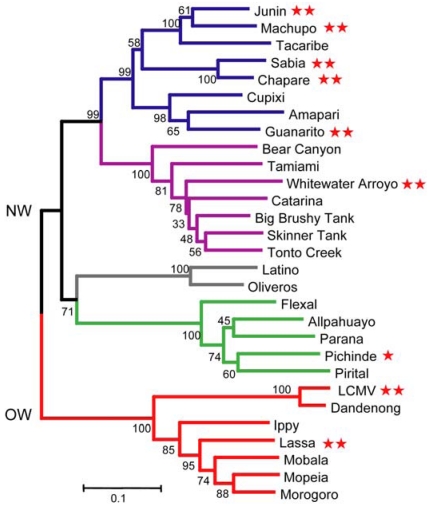
Phylogenetic relationships between arenaviruses. The Old World (OW) virus lineage is depicted in red, and New World (NW) lineages are subdivided into clades: A (green), B (blue), C (gray) and a recombinant A-B lineage (purple). This phylogenetic tree is based on amino-acid comparisons of the GPC. Red stars that follow virus names indicate the ability to infect humans: ** denote lethal hemorrhagic fever viruses and lymphocytic choriomeningitis virus (LCMV), and * identifies infections with few or no clinical manifestations [1]. This image is adapted from [1] and used by permission.

Arenaviruses constitute a diverse family of enveloped negative-strand RNA viruses with worldwide distribution. At least 29 arenavirus species are currently recognized [1], and that number continues to increase as new viruses are identified [2,3]. Arenaviruses are endemic to rodent populations, and have speciated in parallel with their rodent hosts [4,5,6,7]. Of the arenavirus species, over 30% can also infect humans and eight are associated with hemorrhagic fevers and lethal disease (Figure 1) [1]. Lassa fever virus (LASV), an Old World (OW) arenavirus species, is endemic in western Africa and affects 2–3 million persons annually. Five species of New World (NW) hemorrhagic fever viruses—Junín, Machupo, Guanarito, Sabia, and Chapare—are distributed throughout South America. In nature, transmission to humans is typically through direct contact with infected rodents or inhalation of rodent excreta. Instances of person-to-person spread have also been documented, often through nosocomial or familial infection. Lassa fever is occasionally imported to Europe and the United States by infected travelers [8,9]. In the developed world, infection with lymphocytic choriomeningitis virus (LCMV), an OW arenavirus associated with neurological and congenital disease, has been reported in organ transplant recipients and immunocompromised patients [10,11].

Infection by the hemorrhagic fever arenaviruses is accompanied by high morbidity and mortality, with significant sequelae in survivors [12,13]. Therapeutic options are currently limited to the nonspecific antiviral ribavirin, whose use is restricted to high-risk patients due to its adverse effects and mixed efficacy [14]. Several small-molecule compounds that specifically inhibit arenavirus entry have recently been described, and show promise for treatment and post-exposure prophylaxis [15,16,17,18,19]. Human immune plasma containing neutralizing antibodies against JUNV is used for post-exposure protection against Argentine hemorrhagic fever [20]. Antibody-mediated therapy against Lassa fever appears to be more problematic [14].

At present, there are no FDA-licensed vaccines to protect against arenavirus infection and disease. This status likely reflects historical priorities rather than biological impediments. The attenuated Candid#1 strain of JUNV has been shown to provide safe and effective prophylaxis against Argentine hemorrhagic fever, and has been used in more than 200,000 persons in Argentina, but remains unlicensed in the United States [21,22]. The molecular basis for attenuation of the Candid#1 isolate is currently under investigation [23,24]. Recent advances in vaccine technology suggest that protective immunization against Lassa fever may also be possible [25,26,27,28]. In the absence of approved vaccines and effective therapies, however, the hemorrhagic fever arenaviruses are recognized by the United States as Category A priority pathogens for biodefense research [29]. JUNV has further been determined to pose a Material Threat to the US population at large [30].

## 2. The Unusual GPC Envelope Glycoprotein

The arenaviruses are enveloped viruses with a bisegmented negative-strand RNA genome that encodes the expression of four proteins using an ambisense coding strategy [31]. As several excellent reviews offer background information regarding the genomic organization and life cycle of the arenaviruses [14,28], we will focus our discussion on the virus envelope glycoprotein and virus entry. Intervention strategies that target these early events in infection have historically been fruitful in the prevention and treatment of viral disease.

**Figure 2:**
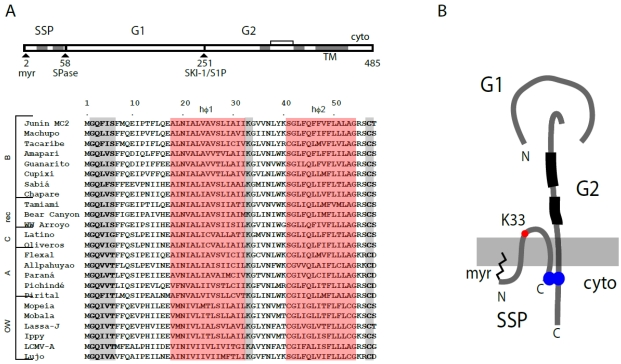
Schematic representation of GPC open-reading frame, stable signal peptide (SSP) sequence alignment and the tripartite GPC protein complex. (**A**) The GPC open‑reading frame is diagrammed. Cleavage sites for signal peptidase (SPase) and subtilisin-like kexin protease-1/site-1-protease (SKI-1/S1P) are indicated, as are the mature SSP, G1 and G2 subunits. In SSP, the myristoylation at glycine 2 is marked, and the shaded regions denote the two hydrophobic regions (hɸ1 and hɸ2, highlighted in red in the sequence comparisons below). The transmembrane and cytoplasmic domains of G2 are indicated, as well as the two heptad‑repeat regions (shaded) and disulfide-bonded hinge region. The sequence comparison of SSP among arenaviruses is adapted from [32], in which accession numbers are listed. In addition to hɸ1 and hɸ2 (red), the conserved myristoylation motif, K33 and C57 residues are highlighted in gray. (**B**) Schematic drawing illustrating the subunit organization of the tripartite GPC complex. The membrane is shown in gray. SSP spans the membrane twice, and salient features are indicated: Membrane association of the myristoylated N-terminus, K33 in the SSP ectodomain, and the intersubunit zinc-binding motif that bridges the C‑terminal cytoplasmic domains of SSP and G2. The two heptad-repeat regions in the G2 ectodomain are depicted in black. The drawing is not to scale and the structural relationships among subunits is not known.

The arenavirus envelope glycoprotein GPC is expressed as a single precursor polypeptide and directed to the endoplasmic reticulum (ER) membrane by its signal peptide (Figure 2A). Following signal peptidase cleavage, the nascent polypeptide is anchored in the ER membrane as a Type I transmembrane protein and oligomerizes to form a trimeric complex. Proteolytic cleavage of this precursor glycoprotein, to generate the mature receptor-binding and transmembrane fusion subunits (G1 and G2, respectively), is required to activate the membrane-fusion potential of the complex. Cleavage is mediated by the site-1-protease (alternatively named subtilisin-like kexin protease-1; S1P/SKI-1) in the early Golgi compartment. The mature noncovalently associated GPC trimer is ultimately trafficked to the plasma membrane, where virion assembly and budding occur.

In contrast to other virus envelope glycoproteins, GPC is unique in that it retains its cleaved signal peptide as a third and essential subunit in the mature complex. The stable signal peptide (SSP) gives rise to a novel tripartite architecture (Figure 2B) with profound repercussions for many aspects of GPC biology. We will return to SSP and its role in GPC-mediated membrane fusion in later sections of this review.

## 3. Cellular Receptors for Arenavirus Entry

Arenavirus entry is initiated by G1 binding to an appropriate cell-surface receptor protein. In the case of the pathogenic NW viruses, G1 recognizes human transferrin receptor-1 (TfR1) [33,34]. Intimate details of this interaction have been revealed through genetic [35,36,37,38] and crystallographic studies [39]. This work demonstrates that G1 binds to the tip of the apical domain of the receptor, distinct from the transferrin-binding site on TfR1 [40]. Comparison of the crystal structure of G1 in the TfR1-bound [39] and unliganded [41] state suggests that TfR1 binding does not induce major conformational changes in the receptor-binding subunit [39]. Therefore, TfR1 likely acts as a passive, albeit critical, attachment point to facilitate virion uptake into the cell.

Interestingly, the genetic and structural analysis of TfR1 binding also reveals the molecular basis for co-evolution of arenavirus species with their respective rodent hosts, and for infection in humans. Phylogenetic analysis divides the arenavirus genus into OW and NW species, and the NW viruses into three clades [42]. Only the clade B viruses, which include the pathogenic New World species, use TfR1 for entry [42]. While all clade B species bind to their rodent-host TfR1, those pathogenic to humans have evolved to additionally recognize human TfR1 [35,36,39]. These host-range restrictions, and the expansion into humans, are determined at the TfR1-G1 interface. A single amino‑acid change in human TfR1 is sufficient to allow entry by a non-pathogenic clade B species (Tacaribe, TCRV) [36]. Conversely, it is possible that only modest changes in G1 of a non-pathogenic virus may be sufficient to enable use of human TfR1 and the emergence of a newly pathogenic species [36]. 

Upon binding to TfR1, the virion is endocytosed and GPC-mediated fusion of the viral and endosomal membranes is activated in response to acidification of the maturing endosome [43,44,45]. This fusion event deposits the virion core into the cytosol to initiate viral replication. As TfR1 is constitutively recycled from the cell surface via clathrin-mediated endocytosis, binding to TfR1 can be imagined to provide an efficient pathway for virion entry. The interaction with TfR1 may also contribute to aspects of pathogenesis in humans [46].

In contrast to clade B viruses, entry by the OW and NW clade C species is dependent on G1 binding to α-dystroglycan (αDG) [47,48,49], a ubiquitous and highly conserved cell-surface glycoprotein involved in adhesion to extracellular matrix. In binding αDG, the OW arenaviruses may bypass the early endocytic pathway through uptake into smooth vesicles [44] and subsequent transport to multivesicular bodies in the late endosome [50,51]. Exposure to the strongly acidic pH in the late endosome may be required to activate membrane fusion by OW GPCs, which are reported to be unusually resistant to low pH [27,51,52]. The cellular receptor utilized for entry by clade A New World viruses is unknown.

## 4. GPC Is a Class I Virus Fusion Protein

On exposure to acidic pH in the maturing endosome, the prefusion GPC complex is activated to undergo a series of conformational changes leading to membrane fusion [43,45]. A generally accepted framework for virus-mediated membrane fusion [53,54] posits that the native envelope glycoprotein exists in a metastable state that is established on proteolytic maturation of the precursor polypeptide. The fusion subunit is sequestered in the prefusion complex and released from its constraints by either receptor binding at the plasma membrane or acidic pH in the endosome, depending on the virus. The activated complex then spontaneously undergoes a structural reorganization leading to formation of a highly stable postfusion structure, and concomitant fusion of the virus and cell membranes. In this series of conformational changes, the ectodomain of the fusion protein is exposed and its hydrophobic fusion peptide is inserted into the target-cell membrane. This intermediate state is subsequently resolved by the collapse of the fusion protein ectodomain to form a stable postfusion structure. In doing so, the ectodomain brings the target and virus membranes into proximity for membrane fusion.

Virus fusion proteins can be subdivided into three classes (I, II and III) based in part on the structure of the postfusion product [53,54]. The arenavirus G2 protein was initially assigned to class I by Gallaher and Buchmeier [55] based on sequence similarities with others in the class, which include the well-studied hemagglutinin of influenza virus, the envelope glycoprotein of human immunodeficiency virus, and the F protein of paramyxoviruses. A defining characteristic of class I fusion proteins is the formation of a highly stable six-helix structure in the trimeric postfusion state. The ectodomains of these proteins contain two stretches of 3–4 repeating hydrophobic residues (N and C heptad-repeats, HRs) that refold during the fusion cascade to form α-helices. Coiled-coil formation is believed to drive insertion of the N-terminal fusion peptide into the target-cell membrane and the subsequent reorganization to form the diagnostic six-helix bundle in which three C helices pack in an antiparallel orientation onto hydrophobic grooves on the surface of a central trimeric N-helix coiled coil. This brings the virus and cell membranes into apposition for membrane fusion. In keeping with this model, Gallaher and Buchmeier showed that a peptide from the N-HR (LCMV GPC residues 326–355) take on helical character in solution [55].

Further evidence for six-helix bundle formation in GPC was derived from biochemical and biophysical studies performed by Eschli and colleagues [56]. By using dynamic light scattering and circular dichroism, these investigators found that an LCMV N-HR peptide (residues 325–354) forms a trimeric α-helical structure in solution, consistent with the central N-helical coiled-coil in the six-helix bundle. While a C-HR peptide (residues 404–428) itself did not show significant helicity, recombinant G2 ectodomain constructs bearing both N- and C-HR regions folded to form a helical trimer consistent with a six-helix bundle. Using an unbiased protein-dissection approach, Lu and colleagues isolated the protease-resistant structure formed on refolding a recombinant G2 ectodomain of JUNV [57]. This stable core was found to contain of N- and C-HR sequences. Circular dichroism spectroscopy and analytic ultracentrifugation showed further that an equimolar mixture of the N- and C-HR peptides (residues 325–353 and 382–411, respectively) form a highly stable (*T*_m_ = 64 °C) trimer of heterodimeric N- and C-helices. (Note that because LCMV and JUNV differ slightly in length, a direct comparison of residue numbers is only approximate). These models of the G2 six-helix bundle were validated in the recent crystal structure of the postfusion ectodomain of LCMV G2, which contains an extended N-helix (residues 316–360) and short C-helix (residues 408–420) [58].

To investigate the functional significance of the presumptive six-helix bundle in GPC, we applied alanine-scanning mutagenesis at predicted interhelical residues throughout the N- and C-HRs of JUNV (***a*** and ***d*** helical positions). Alanine was chosen as it shows good helical propensity but contributes little to the hydrophobic interactions expected to stabilize the coiled coils. Consistent with the six-helix bundle model, alanine substitutions at four positions in N-HR (I333, L336, L347 and L350) and two positions in C-HR (R392 and W395) resulted in specific defects in pH-dependent GPC-mediated membrane fusion [59]. The polar sidechain R392 may impart specificity to the process of coiled-coil folding, at the expense of thermal stability [60]. Taken together, these studies place arenavirus GPC firmly among the class I fusion proteins.

In the current model of membrane fusion, the envelope glycoprotein forms a transient intermediate structure in which the fusion peptide is inserted into the target-cell membrane to bridge the two membranes. The hydrophobic fusion peptide of class I envelope glycoproteins is generated through proteolytic cleavage of the glycoprotein precursor, and generally comprises 15–25 amino acids at or near the N terminus of the fusion subunit. By contrast, the fusion peptides of class II proteins (e.g., flavivirus E and alphavirus E1) and class III proteins (e.g., vesicular stomatitis virus G and herpes simplex virus gB) are located internally and include one or two disulfide-bonded loop regions [53]. Curiously, the fusion peptide of arenavirus G2 appears to combine features from the three classes. Genetic analysis reveals two elements at and near the N terminus of G2 that are critical for membrane fusion [52]. The N-terminal region is relatively hydrophobic but foreshortened by a conserved aspartic acid at position nine. Another fusion peptide domain is located 10–35 amino acids from the N terminus, in a region of short hydrophobic stretches interspersed with charged residues [52]. This internal region includes a series of four cysteine residues that may form one or more disulfide‑bonded loops [57]. A similarly ‘hybrid’ organization has been proposed for the fusion peptide of avian sarcoma/leukosis virus [61].

## 5. The Unusual SSP Signal Peptide

Although the structural changes associated with formation of the postfusion six-helix bundle in GPC may be analogous to those of other Class I fusion proteins, the molecular basis for pH-induced activation of membrane fusion is no doubt distinct, owing to the involvement of the unique SSP subunit.

The first indications that SSP might be more than a conventional signal peptide arose from studies at the University of Marburg [62]. Eichler and colleagues identified the N-terminal residue of the LASV G1 subunit at position 59, and showed that cleavage at the terminal SSP residue (T58) obeyed rules previously established for signal peptidase [63]. Mutagenesis revealed a similar pattern of permitted and unacceptable residues in JUNV GPC [64]. At the prodigious length of 58 amino acids, SSP is also considerably longer than conventional signal peptides (typically 18–30 amino acids). Furthermore, SSP contains two distinct hydrophobic domains [65] rather than the single h-region found in other signal peptides [63]. These features are conserved among all arenavirus species (Figure 2A) and we will refer to NW and OW arenaviruses interchangeably in this discussion.

Another unusual property of the GPC signal peptide noted by Dobberstein and colleagues is that SSP is extraordinarily long-lived in the cell (*t*_1/2_ ~ 6 h) [65]. With the availability of robust monoclonal antibodies [66] and the advent of high-resolution precast gels for SDS-PAGE, we were able to show by co-immunoprecipitation that the small SSP (~5 kDa) was in fact an integral component of the JUNV GPC complex [67]. Thus, unlike other Class I viral fusion proteins, mature GPC comprise three subunits: the unique SSP subunit as well as the canonical receptor-binding and transmembrane fusion proteins. SSP association in the complex depends solely on the transmembrane and cytoplasmic domains of G2 and is not affected by replacing the entire GPC ectodomain with that of an unrelated protein [68,69]. This tripartite architecture invites multiple questions regarding the structure and function of GPC. 

In further contrast to conventional signal peptides, SSP is myristoylated at a conserved N-terminal myristoylation motif [67]. Interestingly, the non-myristoylated G2A mutant is partially defective in its membrane-fusion activity [67,70]. The molecular basis for this defect is unclear [70,71]. Adding to the mystery is the fact that SSP can be expressed separately from the G1G2 precursor (modified to contain a conventional signal peptide) and will associate in *trans* to reconstitute the functional GPC complex [67,72].

Early studies revealed that SSP association was required for proteolytic maturation of the G1G2 precursor [72]. The basis for this requirement is somewhat unresolved. SSP may act as a chaperone to ensure appropriate folding of the GPC precursor for S1P/SKI-1 cleavage [72,73]. Alternatively, our studies suggest that SSP association is required for transport of the G1G2 precursor through the Golgi compartment [68], in which the active form of S1P/SKI-1 resides [74]. Confocal microscopy studies show that in the absence of SSP, the JUNV G1G2 precursor is retained in the ER and that mutations at either of two dibasic ER retention/retrieval motifs near the cytoplasmic C terminus of G2 offer partial relief from the requirement for SSP [68]. In controlling trafficking, SSP may act as a quality-control element to ensure that only the complete tripartite GPC complex is transported to the cell surface for incorporation into budding virions.

The presence of two hydrophobic sequences in SSP raises the question of how SSP is positioned in the membrane. While several topologies have been suggested based on biochemical studies [65,75,76], our work favors the notion that SSP spans the membrane twice with both N and C termini localized to the cytosol [77]. For these studies, we introduced an affinity tag into permissive sites in either the N‑ or C-terminal region of JUNV SSP. These constructs were able to support GPC assembly, processing and transport to the cell surface. By selectively permeabilizing the plasma membrane with digitonin, we could show by confocal microscopy that both the N- and C-terminal tags reside in the cytosol [77]. Thus, the two hydrophobic regions in SSP span the membrane in an antiparallel orientation, and are joined by a short intervening ectodomain loop (Figure 2B). Of note, the myristoylated N terminus remains in the cytosol, as does the C terminus containing a uniformly conserved cysteine residue (C57).

**Figure 3:**
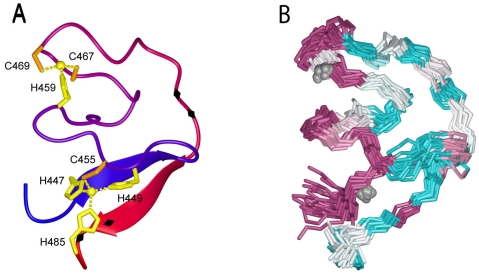
NMR structure of the zinc-binding domain in the cytoplasmic tail of G2. (**A**) Ribbon model of the deduced structure. Coloring changes from blue (N terminus) to red (C terminus). The zinc ions are depicted as spheres, and the coordinating cysteine and histidine sidechains are displayed. Black diamonds on the C-terminal half of the ribbon depict the two pairs of basic residues (KK and RR) implicated in ER retention/retrieval. (**B**) Superimposed backbone traces are colored to indicate the degree of sequence conservation among arenaviruses: highly conserved residues are shown in purple and highly variable residues in cyan.

Initial alanine-scanning mutagenesis revealed that among the four residues in the nominal C‑terminal cytoplasmic domain of JUNV SSP, only C57 was essential for GPC assembly, transport, processing and membrane-fusion activity [64]. Specifically, C57 was required for SSP association in the GPC complex. Sequence analysis subsequently led us to identify an array of six conserved cysteine and histidine residues in the cytoplasmic domain of G2 that were likewise required for SSP association [78]. This array is similar to those found in zinc-binding domains (ZBDs) and equilibrium binding studies using a maltose-binding fusion protein containing the cytoplasmic domain of JUNV G2 confirmed its ability to bind zinc ion with subnanomolar affinity. The three membrane-proximal residues (Zn1: H447, H449 and C455) were found to be required for high-affinity binding, whereas the latter three (Zn2: H459, C467 and C469) appeared to be dispensable. However, as all the cysteine and histidine mutations ablate SSP association, we proposed that the latter three sidechains participated with C57 in SSP to form a second zinc-binding center. This notion was recently confirmed in the structure of the JUNV G2 cytoplasmic domain obtained using NMR spectroscopy [32]. In the absence of SSP, the cytoplasmic domain of G2 folds to display two distinct clusters of cysteine and histidine residues on one face of the molecule, each posed to coordinate zinc (Figure 3). Isothermal titration calorimetry was used to confirm the binding of two zinc ions. By using a tripeptide model for SSP (acetyl-SCT), we could also show that C57 completes coordination at Zn2 to form an intersubunit zinc-finger structure. The zinc-binding face of the cytoplasmic domain is well conserved in amino-acid sequence among arenaviruses (Figure 3). However, the C-terminal portion of G2 containing the dibasic ER retention/retrieval motifs is variable in sequence and wraps around the structure to form the opposite face. The molecular basis by which SSP association masks ER retention/retrieval signals is not apparent from the structure. Taken together, these studies show that SSP is retained in the GPC complex through a novel intersubunit zinc-binding structure that may position it for pH-sensitive interactions with the ectodomain of G2.

## 6. The Role of SSP in GPC-Mediated Membrane Fusion

**Figure 4:**
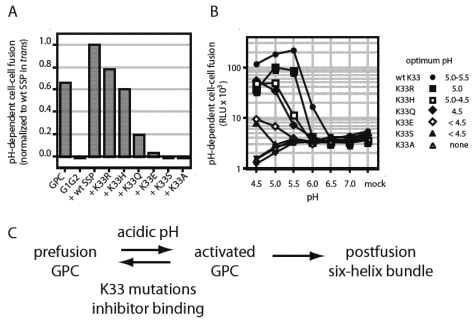
K33 in SSP modulates pH-induced activation of membrane fusion. (**A**) Mutations that reduce positive polarity at SSP K33 were introduced into SSP and the membrane-fusion activity of the GPC complex was determined in our standard assay for cell-cell fusion. All SSPs were expressed in *trans* with the G1G2 precursor to reconstitute GPC, and fusion activity was normalized to that of the wild-type (K33) SSP. GPC and G1G2 indicate, respectively, the complete GPC open-reading frame and the precursor alone, in the absence of SSP. (**B**) The pH-dependence of the mutant GPC complexes was determined by varying the pH of the triggering pulse of acidic medium. The nominal optimal pH for each mutant is listed at right. (**C**) Model for pH-induced activation of GPC, and its inhibition by mutations at K33 and by small-molecule compounds. Acidic pH destabilizes the metastable prefusion GPC complex to favor an activated state, that spontaneously undergoes the class I structural reorganization resulting in formation of the six-helix bundle and membrane fusion. By contrast, K33 mutations and inhibitor binding disfavor the transition to the activated state. The molecular basis for maintaining the equilibrium between prefusion and activated states is unknown.

The first indication that SSP was directly involved in GPC-mediated membrane fusion came from our observation that an alanine substitution at the conserved K33 position in the short ectodomain loop of SSP specifically ablated pH-dependent membrane fusion while sparing GPC biosynthesis, assembly, processing and transport to the cell surface [79]. Conservative mutations (K33R, K33H and K33Q) revealed a systematic loss in fusion activity that correlated with the extent of positive polarity at that position (Figure 4A). Further studies demonstrated that this effect was mediated through a progressive reduction in the pH required to activate fusion, rather than in the inherent fusion potential *per se*. In our assay for GPC-mediated membrane fusion, JUNV GPC-expressing cells are pulsed with low-pH medium to initiate the fusion process. Whereas the wild-type GPC fuses optimally at pH 5.0–5.5, consistent with endosomal entry, fusion by the K33Q mutant requires pH 4.5 (Figure 4B). These studies led us to suggest that the K33 sidechain in the ectodomain loop of SSP participates in pH sensing and pH-induced activation of membrane fusion. Specifically, we propose that K33 participates in maintaining the metastable prefusion GPC at neutral pH, and destabilizing the complex at low pH to activate the Class I fusion cascade (Figure 4C). Mutations that reduce the positive charge at this position stabilize the prefusion state against activation by acidic pH.

As the lysine sidechain at position 33 is not itself affected by low pH, we reasoned that the ectodomain loop of SSP might interact with the membrane-proximal ectodomain for G2 to control pH‑induced activation. Indeed, scanning mutagenesis of conserved charged residues in this region of G2 identified several positions (D400, E410, R414 and K417) that, when mutated to alanine, rescued the pH-dependent defect in the K33Q mutant [80]. Interestingly, similar phenotypes were observed on substituting alanine at several hydrophobic positions within the G2 transmembrane domain (F427, W428 and F438). None of the mutations directly affected the pH of activation, suggesting that rescue was not due to compensating destabilization of the G2 mutants. Additional alanine-scanning mutagenesis identified scattered charged positions throughout G2 that also complemented the K33Q defect (K283, K344 and R348) [81]. Mutations at conserved histidine residues in the G2 ectodomain (H297, H366, and H372) failed to identify residues important for pH-induced activation [81]. While these findings confirm a genetic interaction between SSP and G2, the molecular basis for rescue of the K33Q mutation is not at all apparent. In contrast to the “histidine switch” hypothesis proposed for certain viral envelope glycoproteins [82,83], our findings suggest that pH‑sensing and pH-induced activation in GPC are likely mediated through a highly distributed network of interactions, as has been described for the acid-sensing ion channel protein ASIC1 [84]. 

## 7. Small-Molecule Inhibitors of pH-Induced Activation

**Figure 5:**
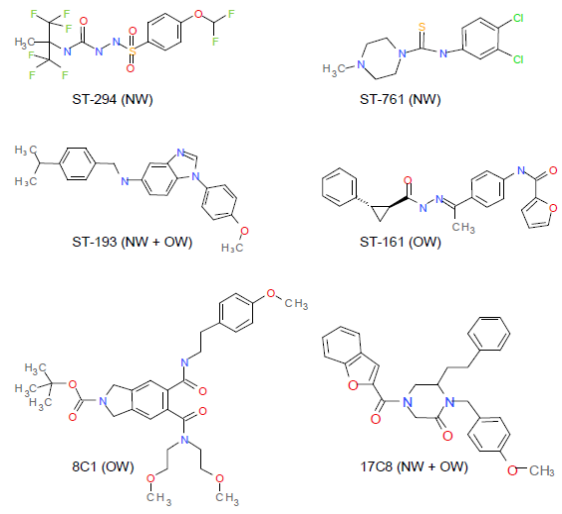
Chemically distinct classes of arenavirus-specific fusion inhibitors. Compounds from SIGA Technologies are indicated with the ST prefix [15,16,85,86] and 8C1 and 17C8 were described at The Scripps Research Institute [17]. All lead compounds were identified through high-throughput screening for inhibition of arenavirus infection or GPC‑mediated entry. The compounds all bind to a common site on prefusion GPC and act through the pH‑sensitive SSP-G2 interface, but differ in their specificities for NW and/or OW arenaviruses (as indicated).

Evidence for the critical role of SSP-G2 interactions in maintaining the prefusion state of GPC and responding to acidic pH can also be deduced from the study of small-molecule arenavirus-specific fusion inhibitors. High-throughput screening programs at SIGA Technologies, Inc. (Corvallis, OR, USA) and the Scripps Research Institute (La Jolla, CA, USA) have discovered at least six chemically distinct classes of compounds that inhibit GPC-mediated membrane fusion [15,16,17,85] (Figure 5). All these inhibitors bind to the prefusion GPC complex, and need only be present prior to exposure to acidic pH in order to act [15,85,86]. Drug-resistant viruses isolated at SIGA were found to contain mutations in the membrane-proximal ectodomain and transmembrane region of G2 [15,16]. Scanning mutagenesis extended these findings and also identified a key locus of resistance at K33 in SSP, reinforcing the notion that SSP and G2 interact to control GPC-mediated membrane fusion [85].

Despite the chemical differences among the inhibitors, genetic and now biochemical evidence suggests that all the classes share a common binding site on GPC. Cross-resistance among the classes is common [16,85] and direct binding measurements demonstrated that all inhibitors active against JUNV compete for binding to JUNV GPC [86]. Interestingly, different classes of inhibitors show different patterns of specificity against NW and/or OW arenavirus species (Figure 5) [15,16,17,85]. The spectrum of activity correlates with binding to GPC [86] and likely reflects sequence divergence within the binding pocket. Consistent with this hypothesis, the K33H mutant of JUNV GPC that is resistant to the NW-specific inhibitor ST-294 shows *de novo* sensitivity to the OW-specific compound ST-161 [85]. Two classes of inhibitors (ST-193 and TSRI 17C8) are active against both NW and OW viruses and may provide lead compounds for the development of broadly active therapeutics to treat arenavirus hemorrhagic fevers.

In sharing a common binding site, the inhibitors likely share a mechanism of action. Genetic studies of inhibitor sensitivity point to some of the same residues that are thought to mediate pH-dependent interactions between SSP and G2, including K33 [80,85]. Indeed, the SIGA inhibitors appear to act similarly to charge mutations at K33 to antagonize the pH-induced transition of the prefusion GPC to an activated state committed to the class I fusion cascade (Figure 4C). As noted for K33H and K33Q mutants, inhibitor binding also lowers the pH at which membrane fusion is activated [85]. Conversely, decreasing the pH of the acid pulse can in some cases abrogate inhibition (e.g., ST-193 is only poorly inhibitory of wild-type GPC at pH 4.5). These effects on activation appear to be additive with those of the K33 mutations. In stabilizing the prefusion form of GPC against pH-induced activation, the inhibitors effectively overcome the low pH in the endosome and thereby prevent virus entry. Several SIGA inhibitors have shown efficacy in protecting against lethal arenavirus infection in small-animal models [15,19] and hold promise towards clinical development for the treatment of arenavirus hemorrhagic fevers.

## 8. Future Directions

While much as been learned about the unusual arenavirus envelope glycoprotein in the past decade, many fundamental questions remain unanswered. (1) How is the tripartite GPC monomer organized, and how are the monomers related in the trimeric complex? (2) What are the structural features that: (i) establish fusion competency on proteolytic maturation of the GPC precursor, (ii) maintain the mature metastable prefusion state at neutral pH, and (iii) promote the transition to membrane fusion at low pH? What are the molecular and atomic requirements for effective interference by small-molecule fusion inhibitors? (3) How do interactions among the nine transmembrane domains of the trimer affect pH-sensing and pH-induced activation by the GPC ectodomain? (4) How does SSP myristoylation and formation of the zinc-binding domain in the cytoplasmic domain of GPC affect intracellular transport, virion assembly and membrane-fusion activity. These questions can now be addressed in the context of virus infection by using the robust reverse-genetics systems developed over past years [87,88,89,90]. Reverse-genetics studies will also be important for understanding the role of GPC in arenavirus pathogenesis [23,88].

High-resolution structural analysis of transmembrane envelope glycoproteins is extremely challenging, and crystallographic studies to date have been limited to soluble fragments. Moreover, the intrinsic metastability of the trimeric envelope glycoprotein complex has frustrated efforts to isolate the prefusion form of the protein. Recent studies in our laboratory have shown the transmembrane GPC complex of JUNV to be exceptionally stable to solubilization and purification as a trimeric complex, and to reconstitution in artificial lipid membranes [86]. It is possible that the unique transmembrane organization of the GPC trimer will provide unique opportunities of the structural analysis of envelope glycoprotein-mediate membrane fusion and its inhibition.
